# Advancements in insulin delivery: the potential of natural polymers for improved diabetes management

**DOI:** 10.3389/fbioe.2025.1566743

**Published:** 2025-04-25

**Authors:** Mohammed Ghazwani, Umme Hani, Ashishkumar Kyada, Suhas Ballal, Bahjat Saeed Issa, Munthar Kadhim Abosaoda, Abhayveer Singh, A. Sabarivani, Subhashree Ray

**Affiliations:** ^1^ Department of Pharmaceutics, College of Pharmacy, King Khalid University, Abha, Saudi Arabia; ^2^ Department of Pharmaceutical Sciences, Marwadi University Research Center, Faculty of Health Sciences, Marwadi University, Rajkot, Gujarat, India; ^3^ Department of Chemistry and Biochemistry, School of Sciences, JAIN (Deemed to be University), Bangalore, Karnataka, India; ^4^ College of Pharmacy, Alnoor University, Nineveh, Iraq; ^5^ College of Pharmacy, The Islamic University, Najaf, Iraq; ^6^ College of pharmacy, The Islamic University of Al Diwaniyah, Al Diwaniyah, Iraq; ^7^ Centre for Research Impact and Outcome, Chitkara University Institute of Engineering and Technology, Chitkara University, Rajpura, Punjab, India; ^8^ Department of Biomedical, Sathyabama Institute of Science and Technology, Chennai, Tamil Nadu, India; ^9^ Department of Biochemistry,IMS and SUM Hospital, Siksha ‘O’ Anusandhan (Deemed to be University), Bhubaneswar, Odisha, India

**Keywords:** insulin delivery, natural polymers, diabetes management, controlled release, oral delivery

## Abstract

Diabetes is a growing global health issue, with millions of people affected by the condition. While insulin therapy is vital for managing both Type 1 and Type 2 diabetes, traditional methods such as subcutaneous injections have notable drawbacks, including pain, discomfort, and difficulty in maintaining stable blood sugar levels. To improve insulin delivery, research is increasingly focused on the use of natural polymers—substances derived from plants, animals, and microorganisms. These polymers, including materials like alginate, chitosan, and hyaluronic acid, have promising properties such as biocompatibility, biodegradability, and the ability to provide controlled, sustained insulin release. By encapsulating insulin in polymers, it is protected from degradation and released in a manner that more closely mirrors the body’s natural insulin production. Furthermore, the development of non-invasive delivery methods, such as oral and transdermal systems, is gaining momentum, offering the potential for more patient-friendly treatment options. This review discusses the role of natural polymers in insulin delivery, examining their mechanisms, types, and current research efforts. It also addresses the challenges that remain in advancing these technologies into practical clinical use, aiming to provide more efficient, comfortable, and effective solutions for diabetes management.

## 1 Introduction

The rising prevalence of diabetes worldwide has made it a major global health challenge. It is reported that over 400 million people are affected by this chronic condition, a number that continues to climb due to demographic changes such as aging populations and lifestyle factors like poor diet and inactivity (Chakravarti and Nag, 2021; Rad et al., 2024). Diabetes can be broadly classified into two types: Type 1 diabetes (T1D), where the body’s immune system destroys insulin-producing beta cells in the pancreas, and Type 2 diabetes (T2D), characterized by insulin resistance and eventual beta cell dysfunction. Both types of diabetes are linked by the need for insulin therapy, but the administration of insulin has its own set of challenges, prompting ongoing research into improved delivery methods (Egan and Dinneen, 2019; Nehru et al., 2023).

Traditional insulin therapy, mainly administered through subcutaneous injections, is effective but has several drawbacks. These include the pain and inconvenience of multiple daily injections, the risk of fluctuating blood glucose levels, and the inability of injections to replicate the body’s natural insulin secretion pattern (Sorli and Heile, 2014). Chronic high blood sugar can cause significant, lasting damage to vital organs like the heart, kidneys, and eyes. It also raises the likelihood of complications, including nerve damage, heart-related issues, and severe conditions like diabetic coma, as well as risks to the fetus in pregnant women, such as birth injuries (Aledan et al., 2023; Rad and Nazeri, 2023). As a result, there has been a significant push in the medical field to develop more advanced methods of insulin delivery that are more efficient, less invasive, and better at mimicking the physiological release of insulin. Ideally, these systems would provide a more precise, continuous, and patient-friendly solution to insulin therapy, improving both the quality of life for diabetic patients and their long-term health outcomes (Ren et al., 2023).

Various methods are being explored to enhance the lives of diabetic patients, such as transdermal insulin delivery, which offers potential benefits like better adherence compared to subcutaneous administration, but faces challenges with inefficient skin absorption and requires techniques like chemical enhancers and microneedles to improve its effectiveness (Zhang et al., 2019). Oral delivery of DNA and genes is also a novel approach for diabetes treatment, providing a less invasive alternative to injections by introducing genes to enhance insulin production or glucose regulation. While it offers improved patient compliance, challenges like DNA degradation in the digestive system and ensuring effective absorption must be addressed through protective coatings and advanced delivery methods (He et al., 2018; Nie et al., 2019). Oral nucleic acid therapy can specifically advantageous in type I diabetes treatment (Nie et al., 2020). Ongoing studies are exploring the automation of insulin delivery systems to replace traditional methods, with closed-loop insulin delivery systems, or artificial pancreases, being a key focus of these efforts (Hovorka, 2011; Nwokolo and Hovorka, 2023).

In this context, natural polymers, such as polysaccharides, proteins, and lipids—are emerging as promising candidates for insulin delivery systems (Mohammadpour et al., 2022). These materials, derived from plants, animals, and microorganisms, offer distinct advantages over synthetic alternatives. Their natural biocompatibility and biodegradability make them more compatible with the human body, reducing the risk of adverse reactions (Xu et al., 2023; Sadat et al., 2024). Additionally, these polymers have the potential to provide controlled, sustained, and targeted release of insulin, addressing some of the limitations of current insulin delivery methods (Devaraji and Jayanthi, 2023). Natural polymers can be engineered into various forms, such as hydrogels, nanoparticles, and microspheres, that encapsulate insulin molecules. This encapsulation protects insulin from degradation and offers a release profile that more closely mimics the body’s natural insulin secretion. For instance, polymers like alginate, chitosan, hyaluronic acid, and tannic acid are being investigated for their ability to control insulin release based on environmental stimuli like pH or ionic concentration (He et al., 2019; Shaikh et al., 2023; Alfatama et al., 2024). This controlled release mechanism helps maintain stable blood glucose levels, potentially reducing injection frequency while improving treatment adherence and patient outcomes. Effectively controlling blood sugar, reduces side effects and significantly increase life expectancy for diabetic patients (Nie et al., 2022).

The idea of using natural polymers for insulin delivery is not entirely new, but recent advancements in the field have opened up new possibilities for non-invasive delivery routes, including oral and transdermal systems. By combining the biocompatible and biodegradable properties of natural polymers with cutting-edge drug delivery techniques such as nanotechnology, it is possible to create more sophisticated systems that offer both effective and patient-centric diabetes management (Shetty et al., 2023; Barfar et al., 2024). These advances hold the promise of revolutionizing the way insulin is delivered, reducing the burden of disease management and enhancing the overall quality of life for millions of individuals living with diabetes.

This article explores the potential of natural polymers for insulin delivery, focusing on the mechanisms that enable controlled release, the types of natural polymers being studied, and the challenges that must be overcome for successful application. We will also review the current state of research, highlighting recent promising findings while addressing the obstacles that remain in bringing these technologies to clinical practice. By examining the properties and potential of these natural materials, we can better understand their role in shaping the future of diabetes treatment.

## 2 Types of natural polymers for insulin delivery

Natural polymers are increasingly being explored for use in drug delivery systems, such as insulin, due to their ability to provide safe, effective, and sustainable therapeutic solutions (Wang X. et al., 2024; Wang Y. et al., 2024). These polymers are derived from natural sources and are recognized for their biocompatibility and biodegradability, making them ideal for medical applications (Fouad et al., 2024).

In addition to insulin, these materials are well-known for their ability to deliver various cargos, including drugs, genes, and other therapeutic agents, to targeted areas in the body. Their versatility and precision make them ideal for a wide range of medical applications (Mao et al., 2001). Notably, these polymers can be utilized to treat acute conditions such as injuries, infections, cancers, and tissue repair, offering a promising approach for localized and effective treatment. By ensuring that therapeutic agents reach the desired location, these materials help to minimize side effects and improve the overall efficacy of treatments. Their potential for enhancing drug delivery makes them valuable in advancing both current and emerging therapeutic strategies (Ramakrishna et al., 2001; Liao et al., 2025).

One of the key advantages of natural polymers is their ability to facilitate controlled release, ensuring that insulin is delivered gradually over time, which helps maintain stable blood glucose levels (Kuna et al., 2024). Furthermore, these polymers can improve insulin stability, protect it from degradation, and allow for more precise targeting to specific areas in the body (Satchanska et al., 2024). As a result, insulin delivery systems based on natural polymers can significantly enhance the management of diabetes. Below, we explore several types of natural polymers that are commonly used in insulin delivery.

### 2.1 Chitosan

Chitosan is a natural, biodegradable polymer derived from chitin, a substance found in the shells of crustaceans like shrimp and crabs. Due to its ability to break down safely in the body, chitosan has become a focus of research in the biomedical field, where its unique properties make it an ideal candidate for a variety of medical applications (Gholap et al., 2024). One of the key areas where chitosan has been extensively studied is tissue engineering. Chitosan’s ability to form scaffolds that support cell growth has made it a valuable material for creating structures that encourage the regeneration of damaged tissues. As the body naturally degrades the chitosan scaffold over time, new tissue continues to form in its place, facilitating the gradual healing of injuries or defects. In addition, chitosan’s properties enhance wound healing by promoting cell migration and regeneration. Its inherent antimicrobial qualities also play a crucial role in reducing the risk of infection, further supporting the healing process (Haider et al., 2024a; Zhang X. et al., 2024). Chitosan is also being investigated for its potential in cancer treatment, particularly in improving the delivery of chemotherapy drugs. By encapsulating these drugs within chitosan-based particles, researchers can protect them from degradation before reaching the target area. This approach ensures that the drugs are released directly at the tumor site, increasing the treatment’s effectiveness while minimizing the side effects commonly associated with traditional chemotherapy (Salahuddin et al., 2024).

Another significant area of interest is chitosan’s role in drug delivery systems. Its ability to encapsulate a variety of substances, such as proteins, small molecules, and hormones, allows for controlled and sustained release of these agents in the body. This controlled release improves the efficacy of drugs by ensuring they are delivered in the right amounts at the right time, leading to more effective treatment outcomes. The versatility of chitosan also makes it a promising material for delivering poorly soluble drugs or those that require protection from the digestive system to remain effective (Almukainzi et al., 2024; Haider et al., 2024b).

One of the most exciting potential applications for chitosan lies in the delivery of insulin for patients with diabetes. Traditional insulin treatments require injections, which can be uncomfortable and inconvenient. However, chitosan’s ability to form a protective barrier around insulin molecules could lead to the development of oral insulin delivery systems. This would allow patients to take insulin in pill form, offering a more convenient and less invasive alternative to injections. Pratap-Singh et al. investigated a novel approach to improve oral peptide delivery, focusing on insulin, by utilizing mercaptonicotinic acid-modified thiolated chitosan (MNA-TG-chitosan). The modified material was designed to enhance mucoadhesion and permeability by disrupting tight junctions between cells. Insulin-loaded nanoparticles (insNPs) were prepared using MNA-TG-chitosan, thiolated chitosan, and unmodified chitosan. The results showed that MNA-TG-chitosan produced smaller nanoparticles, while maintaining insulin encapsulation efficiency and loading. *In vitro* studies demonstrated no cytotoxic effects, with significantly increased insulin uptake by liver cells (HepG2), improved transport across buccal cell layers, and enhanced permeability. These findings suggest that MNA-TG-chitosan holds promise for advancing oral insulin delivery systems (Pratap-Singh et al., 2023). In a different study by Bhattacharyya et al., waste polyethylene terephthalate (PET) was depolymerized into BHET and used to synthesize polyurethane (PU) for oral insulin delivery. PU was combined with alginate (ALG) and chitosan (CS) to create nanoparticles with high insulin encapsulation efficiency and controlled release. These nanoparticles showed long-term hypoglycemic effects and improved insulin bioavailability in diabetic mice, with confirmed safety (Bhattacharyya et al., 2017).

Oral insulin encounters several obstacles in the gastrointestinal tract (GIT), including degradation from stomach acidity, digestive enzymes, and various biological barriers that limit its absorption. Furthermore, insulin’s large size and high water affinity make it difficult to penetrate the intestinal lining, resulting in poor oral bioavailability. To overcome these challenges, researchers have proposed the use of nanocarrier-based drug delivery systems as a potential solution to enhance the effectiveness of oral insulin delivery (Mehrotra et al., 2023). In a study by Pessoa et al., a nanoparticle system for oral insulin delivery was optimized to improve insulin protection and absorption in the GIT. The system utilized chitosan, polyethylene glycol (PEG), and albumin coatings on alginate/dextran sulfate cores. Using response surface methodology, the researchers fine-tuned the formulation, focusing on factors like particle size, polydispersity index (PDI), zeta potential, and insulin release. The optimal formulation, which included 0.03% PEG, 0.047% chitosan, and 1.20% albumin, produced nanoparticles ranging from 313 to 585 nm. This formulation achieved over 45% insulin release within 180 min while maintaining its bioactivity, demonstrating its potential to enhance oral insulin delivery (Pessoa et al., 2023). Seyam et al. developed a new system for delivering insulin orally, targeting the colon, using insulin-loaded pectin-trimethyl chitosan nanoparticles (Ins-P-TMC-NPs). The aim was to enhance bioavailability by taking advantage of the colon’s mild environment, which has lower enzyme activity, a neutral pH, and a longer residence time for the particles. The insulin-loaded nanoparticles were formed by coating trimethyl chitosan particles with pectin. These nanoparticles were evaluated for their size, surface characteristics, insulin release behavior, and structure. *In vitro* tests confirmed they were non-toxic, had efficient cellular uptake, and provided a controlled release of insulin. *In vivo* testing in diabetic rats showed a significant decrease in blood glucose levels, with no evidence of toxicity in histological evaluations. These results suggest that Ins-P-TMC-NPs are a promising approach for effective oral insulin delivery (Seyam et al., 2024).

Physical properties such as size, surface charge, and shape significantly affect the penetration of nanogels in the small intestine. Among these, surface charge is particularly important for mucoadhesion and permeation. Charged particles, especially positively charged ones, are more easily taken up by cells compared to uncharged or negatively charged particles. By taking advantage of the influence of surface charge on nanogel behavior, Wang et al. developed two insulin-loaded nanogels with opposite zeta potentials for insulin delivery: insulin:Carboxymethyl Cellulose Sodium/Chitosan -Nanogels(−) with a negative charge and insulin:Carboxymethyl Cellulose Sodium/Chitosan -Nanogels (+) with a positive charge. *Ex vivo* results showed that the negative nanogels exhibited significantly better adhesion and permeation in the rat jejunum, with adhesion three times higher and permeation 1.7 times higher compared to the positive nanogels. *In vivo*, the negative nanogels resulted in a greater reduction in blood glucose levels, demonstrating better glucose management than the positive nanogels (Wang et al., 2016).

In summary, chitosan stands out as a remarkable material in the biomedical field, with its biodegradability, biocompatibility, and ability to encapsulate and release a range of therapeutic agents. As research into its applications continues, chitosan holds the potential to transform the way we approach treatments in areas such as tissue engineering, cancer therapy, drug delivery, and diabetes management. Its unique properties make it an invaluable tool for improving patient care and advancing medical science. The key points of this section are summarized in Table 1.

**TABLE 1:** Key point of section 2.1.

Key topics	Details
Traditional Insulin Delivery	Insulin is typically administered through injections, which are uncomfortable and inconvenient for diabetic patients.
Chitosan’s Role in Insulin Delivery	Chitosan protects insulin from degradation in the gastrointestinal tract (GIT) and helps in the development of oral delivery systems.
Chitosan Nanoparticles for Insulin Delivery	Chitosan-based nanoparticles are used to encapsulate insulin, offering controlled release and enhancing insulin bioavailability.
Challenges in Oral Delivery	Insulin faces degradation from stomach acids and enzymes, limiting absorption in the GIT, making oral delivery challenging.
Modified Chitosan for Insulin	Modified chitosan, like MNA-TG-chitosan, enhances insulin absorption by improving mucoadhesion and permeability across cell layers.
*In Vivo* Research	Studies with chitosan-based insulin systems show improved glucose control and no cytotoxic effects in animal models.
Chitosan Nanocarriers for Oral Insulin	Chitosan nanocarriers protect insulin in the GIT, helping it reach the small intestine for better absorption and effectiveness.
Future Potential	Chitosan-based systems hold promise for oral insulin delivery, offering a more convenient and non-invasive alternative to injections.

### 2.2 Alginate

Alginate, a naturally occurring polysaccharide extracted from brown algae, has attracted significant interest in the biomedical field, particularly for drug delivery purposes. It is composed of two primary sugar monomers, mannuronic acid and guluronic acid, which form a gel-like structure when exposed to divalent cations such as calcium. This unique property, along with its biocompatibility and biodegradability, makes alginate a promising material for insulin delivery systems, which is crucial in the treatment of diabetes (Abourehab et al., 2022; Astaneh et al., 2024). One of the key advantages of alginate in insulin delivery is its excellent biocompatibility, making it well-tolerated by the human body. This reduces the risk of immune reactions or toxicity, which is especially important for long-term use in conditions like diabetes. Alginate-based systems offer an effective approach for encapsulating insulin, enabling controlled and sustained release over time that closely mirrors the body’s natural insulin secretion pattern (Shetty et al., 2023; Wu et al., 2023).

An essential benefit of using alginate in insulin delivery is its ability to protect insulin from degradation. Insulin is sensitive to harsh conditions in the digestive tract, particularly acidic environments such as those in the stomach, where it can be broken down before it reaches the bloodstream. Alginate’s gel-forming properties can encapsulate the insulin, creating a protective barrier that prevents this degradation and allows the insulin to pass through the GIT intact. This makes oral delivery of insulin a feasible option, which would be a more convenient alternative to daily injections. Wu et al. explore a novel approach to delivering insulin orally through the use of liposome-in-alginate hydrogels. In this technique, insulin is encapsulated in liposomes (Lip) along with arginine (AINS) and then combined with a hydrogel created from cysteine-modified alginate (Cys-Alg). The study demonstrates that this formulation significantly enhances insulin absorption, with intestinal permeability of AINS-Lip being six times greater than that of free insulin. Additionally, the hydrogel formulation slows the release of insulin and improves its retention in the intestinal mucosa. In animal studies, the AINS-Lip-Gel was found to release insulin in a controlled manner, leading to effective blood sugar regulation. This approach presents a promising solution to the challenges of oral insulin administration, particularly concerning bioavailability and absorption (Wu et al., 2023).

Moreover, the controlled release of insulin is a significant advantage of alginate-based delivery systems. Alginate’s gel network can be engineered to release insulin at a steady pace by modulating factors such as the concentration of calcium ions or the molecular weight of the alginate. When delivered subcutaneously, this gel-like material gradually dissolves, releasing insulin in a manner that mimics the body’s natural rhythm. This sustained release reduces the frequency of injections, thus improving patient compliance and making long-term management of diabetes easier. Xia and colleagues engineered calcium alginate microspheres with high sphericity using a double emulsion approach, where sodium alginate formed the inner phase. They controlled the microsphere size and sphericity by varying the flow rate and calcium ion concentration in the collecting solution. In their drug release evaluation, insulin-loaded microspheres reduced blood glucose levels in mice by 41.4%, indicating their potential for targeted, pH-sensitive insulin delivery applications (Xia et al., 2024).

Optimizing alginate-based insulin delivery systems presents several challenges. One major issue is controlling the release kinetics of insulin. While alginate gels can offer controlled release, managing fluctuations in blood glucose levels requires more responsive systems that release insulin based on glucose concentrations. Researchers are exploring ways to modify alginate’s structure, including incorporating glucose-responsive elements for targeted insulin release when blood glucose levels rise. Another challenge is ensuring the stability of alginate in oral delivery, as it may degrade in the stomach’s acidic environment. To overcome this, alginate can be crosslinked or combined with other polymers like chitosan to improve its stability and protect insulin as it travels through the gastrointestinal tract, ensuring effective absorption in the intestines (Ren et al., 2023; Shetty et al., 2023; Wang Y. et al., 2025).

Despite these challenges, research into alginate-based insulin delivery systems continues to make significant progress. Advancements are being made to enhance the mechanical strength of alginate hydrogels, improve the accuracy of insulin release profiles, and incorporate systems that respond dynamically to blood glucose levels. These innovations bring us closer to developing a system that can provide a more personalized and efficient method for insulin delivery. In conclusion, alginate is a highly promising material for insulin delivery due to its biocompatibility, ability to form hydrogels, and controlled release capabilities. Although there are still challenges to address, particularly with respect to release control and stability, ongoing research is making substantial strides in overcoming these obstacles. If successful, alginate-based insulin delivery systems could revolutionize the way diabetes is treated, providing more convenient, efficient, and less invasive options for patients around the world. The key points of this section are summarized in Table 2.

**TABLE 2:** Key point of section 2.2.

Key topics	Details
Material Overview	Alginate is a biocompatible, biodegradable polysaccharide from brown algae, used for insulin delivery
Insulin Protection and Release	Protects insulin from stomach degradation and provides controlled, sustained release for improved blood glucose regulation.
Oral Insulin Delivery	Alginate enables oral insulin delivery by enhancing absorption and protecting insulin in the gastrointestinal tract.
Hydrogels and Microspheres	Liposome-in-alginate hydrogels improve absorption, while calcium alginate microspheres provide gradual insulin release.
Challenges	Challenges include controlling insulin release based on glucose fluctuations and stability in the acidic stomach environment.
Future Potential	Research is focused on improving release control, stability, and glucose-responsive systems for personalized insulin delivery.

### 2.3 Hyaluronic acid

Hyaluronic acid (HA) has garnered attention in the field of drug delivery, particularly for insulin, due to its biocompatibility, biodegradability, and ability to enhance absorption. As a naturally occurring glycosaminoglycan, HA is abundant in various tissues and is known for its hydrophilic nature, which makes it an excellent candidate for controlled release formulations. In the context of insulin delivery, HA has been explored for encapsulating insulin, protecting it from premature degradation and facilitating its release in a controlled manner, especially under the dynamic pH conditions of the gastrointestinal tract (Wang et al., 2023).

HA-based delivery systems offer several advantages for insulin administration, particularly in the development of hydrogel or nanoparticle systems that can regulate insulin release rates. By crosslinking HA, the release of insulin can be controlled to occur gradually in response to environmental factors, such as pH changes between the stomach and intestines. This controlled release provides a more consistent insulin profile, potentially reducing the frequency of injections and improving patient compliance with treatment. In this context, Wang and colleagues developed a nanoparticle system using HA and poly-2-((dimethylamino)ethyl methacrylate) (PDM) for oral insulin delivery. The system featured HA with varying molecular weights (low, medium, and high) and demonstrated insulin release that responded to pH shifts, protecting the drug from degradation and enhancing absorption efficiency. The high molecular weight HA formulation showed significant insulin retention and effectively lowered blood sugar levels in diabetic rats, achieving a bioavailability of 14.62%. These nanoparticles offer a promising approach for oral insulin delivery in industrial applications (Wang et al., 2023). In a different study, Huang et al. created a delivery system using layered double hydroxide (LDH) nanoparticles modified with deoxycholic acid (DCA) and hyaluronic acid (HA) for oral insulin administration. This system showed good compatibility with Caco-2 cells and improved insulin absorption by temporarily opening tight junctions between cells. In diabetic mice, oral delivery of insulin-loaded nanoparticles resulted in a sustained hypoglycemic effect lasting 12 h with minimal fluctuations in blood glucose levels. The nanoparticles also enabled better insulin penetration into epithelial cells than free insulin, with the cholic acid transporter receptor (SLC10A1) identified as a crucial component of the system’s action (Huang et al., 2021).

Additionally, HA plays a crucial role in improving insulin absorption across the gastrointestinal barrier. HA interacts with specific receptors, such as the CD44 receptor, found on the surface of intestinal epithelial cells. This interaction aids in the uptake of insulin, boosting its bioavailability when taken orally. Receptor-mediated transport is especially beneficial for oral insulin delivery, as it enhances insulin sensitivity and helps overcome the limitations associated with traditional subcutaneous insulin administration (Hasib et al., 2019). CD44, a key hyaluronic acid receptor, has various isoforms and modifications that offer therapeutic potential. However, designing HA-based therapies is challenged by limited understanding of CD44’s complexity and off-target effects (Rios de la Rosa et al., 2018; Choi et al., 2019).

Furthermore, combining HA with other biocompatible polymers, such as chitosan or alginate, can improve the stability and release kinetics of insulin. These hybrid systems provide enhanced protection for insulin during gastrointestinal transit and optimize absorption, further increasing the therapeutic potential of insulin. Overall, hyaluronic acid holds significant promise in advancing insulin delivery systems, offering controlled release, enhanced stability, and improved absorption, particularly for oral administration, which could lead to more effective and patient-friendly diabetes management. Wu et al. developed a delivery system for oral insulin using hyaluronic acid-coated chitosan nanoparticles (HCP). These nanoparticles are designed to protect insulin from digestive breakdown, enhance its absorption by intestinal cells, and increase its overall bioavailability. This was demonstrated by improved insulin uptake in Caco-2 cells and an *in vivo* pharmacological availability of 13.8%. The findings highlight the potential of the HCP system as an effective platform for oral insulin delivery, offering a promising approach to overcoming the challenges associated with traditional insulin administration methods (Wu et al., 2022). The key points of this section are summarized in Table 3.

**TABLE 3:** Key point of section 2.3.

Key topics	Details
Properties of the Material	HA is a biocompatible, biodegradable glycosaminoglycan, known for its hydrophilic nature, which aids in controlled insulin release.
Controlled Release	HA-based systems allow gradual insulin release, particularly responsive to pH changes in the gastrointestinal tract.
Nanoparticle Systems	HA nanoparticles, such as those combined with PDM or LDH, improve insulin retention, absorption, and bioavailability.
Receptor Interaction	HA enhances insulin uptake through interaction with CD44 receptors on intestinal epithelial cells, improving bioavailability.
Hybrid Systems	Combining HA with polymers like chitosan or alginate improves insulin protection, stability, and absorption during GI transit.
Therapeutic Potential	HA-based delivery systems, like HA-coated chitosan nanoparticles, enhance insulin delivery efficiency and offer a promising alternative to traditional injections.

### 2.4 Gelatin

Gelatin is a natural biopolymer that has gained significant attention in insulin delivery due to its ability to form stable, biocompatible, and biodegradable systems that protect insulin from degradation and enable controlled release. This versatility makes gelatin an ideal candidate for both oral and injectable insulin therapies, which are essential for managing diabetes effectively. Since insulin is a protein hormone, it is prone to enzymatic breakdown in the gastrointestinal tract when taken orally, presenting challenges for this delivery route. Gelatin-based delivery systems overcome these challenges by encapsulating insulin in nanoparticles or hydrogels, which not only protect the hormone from degradation but also provide a steady release, ensuring a more consistent supply of insulin (Taylor et al., 2020; Kumar et al., 2023).

Gelatin nanoparticles are particularly useful for insulin delivery as they can encapsulate the hormone and protect it from the digestive environment. These nanoparticles can be engineered to release insulin gradually, mimicking the body’s natural insulin release patterns (Akhavan Farid et al., 2020). This slow-release mechanism helps in reducing the frequency of insulin administration and maintaining a more stable blood glucose level. Gelatin’s ability to form these nanoparticles provides a robust protection against stomach acids and digestive enzymes, ensuring a larger proportion of insulin reaches the intestine for absorption. Once in the intestine, the insulin can be taken up by the epithelial cells and enter the bloodstream, where it is needed to regulate glucose levels (Jana et al., 2023).

In addition to nanoparticle formulations, gelatin can also be used to create microparticles or microparticles-based systems for controlled insulin delivery. When insulin is encapsulated in gelatin microparticles, these particles act as a shield, ensuring that insulin is not degraded before it reaches the intended absorption site. Moreover, the gelatin microparticles can be designed to release insulin at a controlled rate over time, reducing the fluctuations in blood glucose levels and providing a more consistent therapeutic effect. In a study by Goswami et al., gelatin nanoparticles were synthesized and characterized, focusing on their swelling behavior in PBS under varying conditions like pH, temperature, and chemical composition. Insulin-loaded nanoparticles exhibited controlled release profiles, with factors such as drug loading, nanoparticle composition, and environmental conditions impacting the release rate. The results indicated that the nanoparticles preserved insulin stability, even under highly acidic conditions, highlighting their potential for effective oral insulin delivery (Goswami et al., 2009).

Another promising approach is the use of gelatin hydrogels in insulin delivery. These hydrogels are created by crosslinking gelatin molecules, forming a gel-like matrix capable of encapsulating insulin. The gel matrix controls the release of insulin over time, making it possible to tailor the release profile to match specific therapeutic needs. The degree of crosslinking, the concentration of gelatin, and the addition of other materials can all influence the rate of insulin release, allowing for the creation of fast-acting or long-acting insulin formulations (Zeng et al., 2021). In a recent study by Yerlan et al., the effects of hydrogel matrix composition and crosslinking agents on insulin immobilization were investigated. The team used alginate and alginate–gelatin matrices, crosslinked with CaCl2 and glutaraldehyde. SEM and FTIR analysis showed that beads crosslinked with glutaraldehyde exhibited improved encapsulation efficiency and stability, with gelatin playing a crucial role in preventing insulin fibrilization. This effect was mainly attributed to the dipole-dipole interactions between gelatin and insulin. The findings were further supported by MM2 simulations, which provided deeper insights into the molecular interactions and stability of insulin within the gelatin-based hydrogel matrices (Yerlan et al., 2024).

The biocompatibility of gelatin is a major advantage, as it is well-tolerated by the body and does not cause any harmful side effects when used in drug delivery. Gelatin is also biodegradable, meaning that once it has delivered its insulin payload, it is broken down and eliminated from the body without accumulating in tissues. This property makes gelatin-based systems particularly useful for injectable insulin therapies, where the carrier material does not pose long-term risks or require surgical removal. The ability of gelatin to break down safely and naturally is a significant benefit compared to other synthetic polymers, which may present toxicity concerns (Yuan et al., 2023).

One of the key benefits of using gelatin in insulin delivery is its versatility in formulation. Researchers can manipulate the physical and chemical properties of gelatin-based delivery systems to suit specific patient needs. For instance, insulin-loaded gelatin nanoparticles or hydrogels can be tailored to provide fast or slow release, allowing for better control of blood glucose levels (Bahoor et al., 2023). The tailored release of insulin through gelatin-based systems ensures that it is delivered in a manner that closely mimics the body’s natural insulin production and secretion, providing a more personalized approach to diabetes treatment. Gelatin’s ability to form gel networks and encapsulate insulin also makes it suitable for other drug delivery applications. The gel matrix not only protects insulin but also shields other sensitive drugs from degradation by digestive enzymes or environmental factors. This property broadens the potential of gelatin-based systems, making them useful for delivering proteins, peptides, and biologics that need protection during administration (Qi et al., 2017; Fatima et al., 2024; Ghane et al., 2024).

In summary, gelatin presents a highly promising material for insulin delivery systems. Its biocompatibility, biodegradability, and ability to form nanoparticles and hydrogels make it an excellent candidate for both oral and injectable drug delivery (Zhong et al., 2024). The use of gelatin-based systems ensures the protection of insulin from degradation, provides controlled release, and can be tailored to individual patient needs. With its natural origin, versatility in formulation, and minimal side effects, gelatin is poised to play a significant role in advancing insulin delivery technologies and improving the management of diabetes. Key aspects of this section are summarized in Table 4.

**TABLE 4:** Key point of section 2.4.

Key topics	Details
Material’s Properties	Gelatin is biocompatible, biodegradable, and forms stable nanoparticles and hydrogels that protect insulin from degradation.
Insulin Protection	Encapsulates insulin, shielding it from digestive enzymes and acidic conditions in the GI tract, ensuring safe passage to the intestine.
Controlled Release	Gelatin-based systems (nanoparticles, hydrogels, microparticles) allow for gradual insulin release, mimicking natural insulin secretion.
Formulation Flexibility	Gelatin systems can be tailored for fast or slow insulin release, improving glucose control and providing a personalized approach.
Biocompatibility and Safety	Gelatin is well-tolerated by the body, biodegradable, and does not cause harmful side effects, making it suitable for injectable therapies.
Versatility in Drug Delivery	Beyond insulin, gelatin can be used for other biologics, proteins, and peptides that require protection from degradation.

## 3 Mechanism of insulin release from natural polymers

The controlled release of insulin from natural polymers is a critical area of study for advancing drug delivery systems, especially in managing diabetes. Insulin, a peptide hormone that regulates blood sugar levels, requires careful administration to prevent dangerous fluctuations. Natural polymers are being explored for their potential to develop insulin delivery methods that replicate the body’s natural hormone release patterns. Their biocompatibility and biodegradability make them ideal for creating systems that release insulin in a steady, regulated manner (García-González et al., 2024; Wang Y. et al., 2024). Natural polymers like chitosan, alginate, gelatin, cellulose, and pectin are preferred in these systems due to their non-toxic properties and the fact that they can naturally break down without harming the body. These polymers can also be engineered to control insulin release over time, ensuring that it’s gradual and predictable. Various mechanisms, such as diffusion, swelling, degradation, and external stimuli, govern how insulin is released from these polymers, each contributing to the precise control of release rates.

One common method for insulin release is diffusion-based, where insulin is contained within a polymer matrix. Over time, the insulin gradually moves out of the polymer and enters the bloodstream. The release rate is influenced by factors such as the polymer’s porosity, the size of the insulin molecules, and the polymer’s ability to absorb water. Looser polymer networks typically allow for a faster release. (Ostróżka-Cieślik et al., 2021). Another approach is swelling-controlled release, which is particularly effective with hydrophilic polymers like chitosan and alginate. When exposed to water, these polymers expand, forming channels through which insulin is gradually released. Adjusting the chemical composition of the polymer or altering environmental conditions like pH or temperature can fine-tune the release rate. For example, alginate can swell in the intestine’s alkaline environment, ensuring insulin is released at the right site (Mansoor et al., 2021).

Degradation-controlled release is also a key mechanism for some polymers, such as gelatin and pectin. These materials slowly break down in the body due to specific pH levels or enzymes, releasing insulin over time. The degradation process can be controlled by altering the polymer’s structure or introducing crosslinking, which influences how quickly the polymer breaks down (Sultan et al., 2020; Ali et al., 2021). Ionotropic gelation, which involves creating gel structures by interacting natural polymers with ions like calcium, is another method used in insulin delivery systems. The polymer gels encase the insulin, and as the gel dissolves or reacts with environmental conditions, the insulin is released gradually (Distler et al., 2020). Moreover, stimulus-responsive polymers have been designed to release insulin in response to specific changes, like pH or temperature fluctuations. Some systems react to the temperature of the body, releasing insulin at targeted temperatures, while others are responsive to pH shifts in different areas of the gastrointestinal tract, simulating the body’s natural insulin release (Ali et al., 2021).

In conclusion, insulin release from natural polymers operates through diffusion, swelling, degradation, and external stimuli-responsive mechanisms, each contributing to a controlled and sustained delivery. Ongoing research aims to enhance the efficacy of these systems for diabetes treatment, reducing the need for frequent injections and improving overall blood glucose management.

## 4 Challenges and limitations

Natural polymers, such as chitosan, alginate, hyaluronic acid, and gelatin, are gaining attention for their potential in controlled insulin delivery systems. Their favorable characteristics, including biocompatibility, biodegradability, and the ability to be modified for targeted drug release, make them promising alternatives to synthetic materials. These properties are particularly valuable in drug delivery applications, where patient safety and environmental sustainability are crucial considerations (Nur and Vasiljevic, 2017; Eivazzadeh-Keihan et al., 2024; Zhang H. et al., 2024). However, despite their promising features, several challenges and limitations arise when using these natural polymers in the development of insulin delivery systems.

Chitosan, a polymer derived from chitin, is known for its ability to form gels and its capacity to encapsulate active compounds like insulin. This biopolymer is biodegradable and non-toxic, making it an attractive candidate for medical applications. However, one of the main challenges with chitosan is its limited solubility at physiological pH (Suryani et al., 2024). At neutral pH, chitosan becomes insoluble, which reduces its potential for oral insulin delivery. For chitosan to be effective in the human body, it often needs to be chemically modified to improve its solubility. Despite this modification, chitosan still has a relatively low insulin-loading capacity, limiting the dose of insulin that can be delivered in a single formulation (Elella and Kolawole, 2024). Moreover, the release profile of insulin from chitosan-based systems is often unpredictable. The insulin release rate can vary based on factors like pH and the ionic environment, making it challenging to achieve the desired controlled release. Another issue with chitosan is its rapid degradation in acidic environments, such as the stomach, which complicates its use in oral insulin delivery systems (Ali et al., 2024).

Alginate, a naturally occurring polysaccharide derived from brown seaweed, is commonly used for insulin encapsulation and controlled release. When exposed to divalent cations like calcium, alginate forms hydrogels, making it an ideal material for encapsulating insulin. However, a major limitation of alginate is the instability of its gels under acidic conditions. In the stomach’s acidic environment, alginate can degrade quickly, compromising its ability to protect insulin from premature release before it reaches the intended absorption site in the intestine. This issue can, however, be addressed through proper functionalization of the alginate to enhance its stability and ensure effective insulin delivery (Wong et al., 2011). Additionally, the mechanical properties of alginate gels are often weak, and the gel strength can be highly dependent on the concentration of calcium used in the formulation. Inconsistent gelation results in unstable formulations with erratic insulin release profiles. The viscosity of alginate gels can also pose challenges, particularly for injectable systems, where high viscosity may interfere with the ease of injection and affect the uniformity of insulin delivery (Ramdhan et al., 2020; Raus et al., 2021).

Hyaluronic acid, a polysaccharide naturally found in connective tissues, has garnered attention for its potential in insulin delivery due to its excellent biocompatibility and biodegradability. It has the ability to form hydrogels that can encapsulate drugs like insulin. However, one of the primary challenges with hyaluronic acid is its rapid enzymatic degradation by hyaluronidase, an enzyme that is present in many tissues. This can lead to premature degradation and an unpredictable release of insulin, which is undesirable for controlled drug delivery (Soozanipour et al., 2022; Oliveira, 2023). Additionally, hyaluronic acid typically has a lower insulin-loading capacity than other materials, which restricts the amount of insulin that can be delivered in a single dose. The polymer’s high viscosity may also cause difficulties in formulating injectable systems, as it requires higher injection pressures, making it less convenient for patients to administer. Furthermore, hyaluronic acid’s relatively short half-life in the body means that it is may not ideally suited for sustained insulin release over extended periods, which is essential for managing blood glucose levels effectively (Maleki et al., 2007).

Gelatin, a protein derived from collagen, has been explored for insulin delivery due to its ability to form thermosensitive hydrogels. It is biodegradable and can be processed easily for drug encapsulation. However, gelatin presents challenges due to its sensitivity to temperature. Its physical properties are altered when exposed to higher temperatures, which can compromise the integrity of insulin-loaded systems and lead to the premature release of insulin. Furthermore, gelatin-based delivery systems are often, which increases the likelihood of breakage during injection or while in the body. This brittleness can cause inconsistent release of insulin, which reduces the overall therapeutic efficacy. Another issue with gelatin is that its degradation products may trigger immune responses, particularly if it is derived from animal sources, such as bovine or porcine collagen. This potential for immunogenicity can complicate the use of gelatin in insulin delivery, particularly for patients with sensitivities or allergies to animal-derived products (Ge et al., 2018; Zhu et al., 2018; Madkhali, 2023).

In addition to the unique challenges presented by each of these natural polymers, there are several common limitations that affect the development of insulin delivery systems based on natural materials. One such challenge is the variability in the stability of natural polymers under physiological conditions. These materials are often sensitive to changes in pH, temperature, and moisture, which can lead to their degradation over time (Xie et al., 2017). This degradation can result in the release of insulin in an uncontrolled manner, either too rapidly or too slowly, which undermines the desired therapeutic effect. Achieving a reliable and consistent release profile remains a significant obstacle in the use of these natural polymers for insulin delivery. Furthermore, scaling up the production of insulin delivery systems based on natural polymers presents challenges due to variability in raw materials and manufacturing processes. Batch-to-batch consistency can be difficult to maintain, which can affect the quality and reliability of the final product (Seyam et al., 2020; Limenh, 2024).

Another important limitation is the potential for the natural degradation products of these polymers to interfere with insulin release or trigger immune reactions. As natural polymers break down in the body, they may release by-products that can cause inflammation or immune responses. This can lead to complications and reduce the safety of the insulin delivery system. To address this issue, researchers are focusing on developing more stable polymeric systems that degrade more predictably and do not release harmful by-products (Zhu et al., 2018).

Despite these challenges, natural polymers such as chitosan, alginate, hyaluronic acid, and gelatin continue to show significant promise for insulin delivery. Ongoing research is focused on overcoming the limitations related to insulin loading capacity, release profile stability, and material degradation. Modifications to these natural polymers, such as cross-linking or hybridization with other materials, are being explored to improve their properties and enhance their performance as insulin delivery systems. Advances in nanotechnology and the use of multifunctional polymers are also helping to overcome some of these challenges, offering more precise control over drug release and better stability. Ultimately, while significant hurdles remain, the development of natural polymer-based insulin delivery systems is an exciting area of research. By overcoming the current limitations, these biocompatible and biodegradable materials could pave the way for more efficient, patient-friendly insulin therapies in the future, offering improved blood glucose management for individuals with diabetes.

## 5 Future directions

The development of natural polymer-based insulin delivery systems represents a promising frontier in diabetes treatment, offering solutions that address both the efficacy and convenience of insulin therapy. Natural polymers, including chitosan, alginate, and hyaluronic acid, have garnered significant interest due to their biocompatibility, biodegradability, and versatility in controlling drug release. As the global prevalence of diabetes continues to rise, the need for effective, patient-friendly delivery systems becomes increasingly critical. Despite the considerable advancements made in polymer-based systems for insulin delivery, there remains a strong need for further research, especially in areas such as responsive drug delivery, novel carrier development, and scalable manufacturing techniques.

One area that has shown significant promise is the use of stimuli-responsive natural polymers. These polymers can be engineered to release insulin in response to specific environmental changes, such as pH, temperature, or ionic strength. By closely mimicking the body’s natural insulin release mechanisms, such systems offer the potential for more precise control over blood glucose levels (Vegad et al., 2023). This responsiveness could allow for the reduction in insulin administration frequency, making insulin therapy more manageable for patients. Among the various stimuli-responsive systems, pH-sensitive polymers stand out due to their ability to exploit the varying pH levels found throughout the GIT. For example (Khatibi et al., 2024), these pH-sensitive polymers can encapsulate insulin, releasing it in a controlled manner as the environment changes within the digestive system. The development of such systems is particularly crucial for oral insulin delivery, which faces significant challenges, including enzymatic degradation and poor absorption in the stomach. By controlling the release of insulin in alignment with the digestive process, these systems promise to enhance the stability and bioavailability of insulin when taken orally, improving therapeutic outcomes for diabetic patients (Li et al., 2022).

Beyond pH-sensitive polymers, temperature-sensitive materials are also under investigation for insulin delivery. Polymers like poly(N-isopropylacrylamide) (PNIPAM) undergo phase transitions in response to temperature changes, offering another mechanism for controlled drug release. These temperature-sensitive polymers could be programmed to release insulin in response to slight variations in body temperature, providing a flexible and dynamic approach to insulin delivery that could adjust to the patient’s individual needs. This capability could be particularly beneficial for personalized insulin therapy, ensuring that insulin is delivered when and where it is most needed, and offering a new dimension in managing blood glucose fluctuations (Luo et al., 2023; Salehi et al., 2023).

In addition to stimuli-responsive systems, the development of polymeric nanoparticles has emerged as a promising strategy to enhance insulin bioavailability and absorption. Polymeric nanoparticles composed of biopolymers like hyaluronic acid, chitosan, and alginate have been shown to improve insulin stability by encapsulating the hormone in a protective shell (Ghasemi et al., 2021). This encapsulation protects insulin from enzymatic degradation, while also allowing for sustained release over time. The small size of nanoparticles allows them to be absorbed more easily by the body, enhancing the bioavailability of insulin when administered via oral or transdermal routes (Wang Y. et al., 2024). Surface modifications of these nanoparticles can further improve their ability to target specific receptors in the GIT or skin, increasing the efficiency of insulin uptake and reducing systemic side effects. For example, surface-functionalized nanoparticles can bind to receptors found in the intestinal epithelium, facilitating insulin transport across the epithelial barrier. This approach could significantly improve the effectiveness of oral insulin delivery, which has traditionally been limited by poor absorption and degradation in the digestive system. Moreover, the potential for using nanoparticles for transdermal insulin delivery is an exciting development. Transdermal delivery systems, which deliver insulin directly through the skin, bypass the GIT entirely, offering an alternative route of administration that could be both more convenient and less invasive than subcutaneous injections (Bercea and Lupu, 2024). Research into nanoparticle-based transdermal insulin systems has shown that nanoparticles can penetrate the skin and deliver insulin directly into the bloodstream, providing a non-invasive method of insulin administration that could improve patient compliance and comfort (Wang et al., 2021; Lv et al., 2025).

However, despite the promising advances in polymeric insulin delivery systems, there are still significant challenges in scaling up production to meet clinical demand. The these systems at a commercial scale have hindered their widespread use (Sabbagh et al., 2022). This presents a major barrier to translating laboratory successes into real-world applications. To overcome this, research is increasingly focused on biomanufacturing technologies that can streamline production processes and reduce costs. Techniques such as microfluidic devices allow for precise control over the synthesis of polymeric nanoparticles and hydrogels, enabling the large-scale production of uniform and high-quality delivery systems (Han et al., 2022). Additionally, the automation of encapsulation processes has the potential to improve the consistency and quality of the products, ensuring that each batch meets the required standards for clinical use (Zhang et al., 2024). Further, the integration of green chemistry practices into the manufacturing process can reduce the environmental impact and cost of production, making these systems more sustainable and economically viable (Wang C. et al., 2025).

While the preclinical studies on natural polymer-based insulin delivery systems are promising, it is crucial that long-term clinical trials are conducted to evaluate the safety, efficacy, and biocompatibility of these systems. The transition from laboratory research to clinical applications requires extensive investigation into the long-term effects of repeated insulin administration using polymeric delivery systems. Although these systems are designed to be biocompatible and biodegradable, it is important to assess any potential adverse effects that may arise from prolonged use. Long-term studies will also be necessary to establish regulatory standards for the clinical use of these systems and ensure that they meet the safety requirements for widespread use (Wang Y. et al., 2024).

In conclusion, the future of natural polymer-based insulin delivery systems is incredibly promising, with advances in stimuli-responsive polymers, nanoparticle carriers, scalable manufacturing, and personalized medicine paving the way for more effective and patient-friendly diabetes treatments. While challenges remain in scaling up production and ensuring long-term safety, ongoing research is likely to overcome these barriers, leading to the development of insulin delivery systems that are not only more efficient but also more accessible for patients. With further innovation in materials science, biomanufacturing, and personalized medicine, natural polymer-based systems could revolutionize the way diabetes is managed, offering new hope for improved treatment outcomes and a better quality of life for millions of individuals worldwide.

## 6 Conclusion

Diabetes has become a significant health concern worldwide, impacting millions of individuals. Insulin therapy plays a crucial role in managing both Type 1 and Type 2 diabetes, but traditional methods, especially subcutaneous injections, present challenges such as pain, discomfort, and difficulty in achieving stable blood sugar control. As a result, there is increasing interest in exploring the potential of natural polymers for enhancing insulin delivery. These materials, which are sourced from plants, animals, and microorganisms, offer unique advantages including biocompatibility, biodegradability, and the ability to deliver insulin in a controlled and sustained manner. Encapsulating insulin in these natural polymers helps protect the hormone from premature degradation, allowing for a more consistent and gradual release that better mirrors the body’s natural processes. Additionally, there is significant progress in the development of non-invasive delivery systems, such as oral and transdermal methods, that could provide more accessible and comfortable treatment options. This review examines the role of natural polymers in insulin delivery, exploring their mechanisms of action, various types, and the latest advancements in research. It also discusses the challenges that still need to be addressed to ensure the successful implementation of these technologies in clinical practice, ultimately aiming to offer more effective and patient-friendly approaches to managing diabetes.
